# Guiding Device for Precision Grafting of Peripheral Nerves in Complete Thoracic Spinal Cord Injury: Design and Sizing for Clinical Trial

**DOI:** 10.3389/fneur.2018.00356

**Published:** 2018-05-22

**Authors:** Arvid Frostell, Per Mattsson, Mikael Svensson

**Affiliations:** ^1^Department of Clinical Neuroscience, Karolinska Institutet, Stockholm, Sweden; ^2^Department of Breast, Endocrine and Sarcoma Tumors, Karolinska University Hospital, Stockholm, Sweden; ^3^Department of Neurosurgery, Karolinska University Hospital, Stockholm, Sweden

**Keywords:** spinal cord injury, regeneration, medical device, computer simulation, morphometry, segmental diameter

## Abstract

**Background:**

In an effort to translate preclinical success in achieving spinal cord regeneration through peripheral nerve grafts, this study details the design and sizing of a guiding device for precision grafting of peripheral nerves for use in a clinical trial in complete (AIS-A) thoracic spinal cord injury (SCI). The device’s design and sizing are compared to a simulation of human spinal cord sizes based on the best available data.

**Methods:**

Spinal cord segmental sizes were generated by computer simulation based on data from a meta-analysis recently published by our group. Thoracic segments T2–T12 were plotted, and seven elliptical shapes were positioned across the center of the distribution of sizes. Geometrical measures of error-of-fit were calculated. CAD modeling was used to create cranial and caudal interfaces for the human spinal cord, aiming to guide descending white matter tracts to gray matter at the caudal end of the device and ascending white matter tracts to gray matter at the cranial end of the device. The interfaces were compared qualitatively to the simulated spinal cord sizes and gray-to-white matter delineations.

**Results:**

The mean error-of-fit comparing simulated spinal cord segments T2–T12 to the best elliptical shape was 0.41 and 0.36 mm, and the 95th percentile was found at 1.3 and 0.98 mm for transverse and anteroposterior diameter, respectively. A guiding device design was reached for capturing the majority of corticospinal axons at the cranial end of the device and guiding them obliquely to gray matter at the caudal end of the device. Based on qualitative comparison, the vast majority of spinal cord sizes generated indicate an excellent fit to the device’s interfaces.

**Conclusion:**

A set of SCI guiding devices of seven sizes can cover the variability of human thoracic spinal cord segments T2–T12 with an acceptable error-of-fit for the elliptical shape as well as guiding channels. The computational framework developed can be used with other medical technologies involving the human spinal cord where exact sizes and positioning are of importance.

## Introduction

The spinal cord is the main structure involved in neural signaling between the body and the brain. A spinal cord injury (SCI) typically leads to significant lifelong functional deficits in patients. In thoracic SCI, the loss of axonal connection between the brain and the lumbar spinal cord gray matter is the dominant reason for loss of neurological function. Many promising approaches are in different phases of development, but none has reached clinical routine to date (1).

Elongation of central nervous system (CNS) axons through peripheral nerve system (PNS) grafts has been described for a century (2), but growth into the CNS and reestablishment of functional connections have been difficult to achieve. Rerouting of axons sprouting in PNS grafts to gray matter, which was shown to be more permissive to axon growth than white matter, has reportedly resulted in the reestablishment of functional connections (3, 4). A more recent advance includes positioning the PNS grafts in a biodegradable device to facilitate microsurgical placement of nerve grafts and achieve the slow release of a growth factor (5, 6). This strategy of a device-guided precision grafting has been shown to induce axonal regeneration and functional reconstitution of complete thoracic SCI in rodents.

To apply the concept of guiding regenerating axons through autologous peripheral nerve grafts in a biodegradable device in an SCI patient, the glial scar would have to be excised and replaced with the peripheral nerves and guiding device. Excision of a segment of the spinal cord (cordectomy) has been performed for the treatment of syringomyelia in chronic complete SCI with good results. For malignant spinal tumors, see Konar et al. for a recent overview (7). Performing a cordectomy for the implantation of peripheral nerve grafts in a guiding device is naturally an invasive procedure and can only be considered in a patient with a chronic complete SCI. However, the literature indicates that the cordectomy itself is not associated with the deterioration of neurological function and may even be beneficial for some common clinical problems in SCI patients such as neurogenic pain and spasticity (7).

The design and sizing of a biodegradable guiding device are a major technical obstacle for clinical adaptation of this device for SCI patients. Effective sizing and design requires a detailed knowledge of the length of an SCI, appreciation of the size and variability of the human spinal cord, a map of the white-to-gray matter delineation of the human spinal cord, and information about spinal cord tracts in humans.

The length of an SCI can be determined with magnetic resonance imaging (MRI). In an earlier study by our group, we defined a protocol for combined MRI and intercostal neurophysiology that served as both an anatomical and a functional measurement of the length of an individual SCI (8). Furthermore, to determine the exact segmental cross-sectional diameter of the spinal cord and its variability, we gathered and synthetized published data in a recent review and meta-analysis (9).

In the chronic phase of SCI, the spinal cord cross-sectional area (SCA) at the C2 vertebral level (cranial to the injury) has been shown by several authors to decrease (10–13). The decrease in SCA has been measured between 11 and 30% and correlates with the severity of injury. The vast majority of patients investigated in these studies had cervical injuries; in the only study that included a significant number of thoracic injuries (13), cervical injuries decreased more than thoracic injuries in SCA compared to controls.

White-to-gray matter delineation of the human spinal cord is readily available through representative sections published in the literature (14, 15). However, data on the white matter tracts of the human spinal cord are limited compared to similar knowledge in experimental animals such as rats or mice. Even state-of-the-art textbooks like the *Paxinos Atlas* show schematic representations of the tracts (16). Data on spinal tracts in humans are derived from postmortem anatomical studies of degeneration of certain spinal cord tracts in patients suffering from a cerebral infarction in the motor cortex within a few weeks of death (17) or in patients suffering from sharp trauma to the cord with partial injury and partial loss of function (e.g., Brown–Séquard syndrome). To date, MRI with diffusion tensor imaging and tractography does not have the resolution required for generating high-quality evidence of spinal cord tracts at the same level as anatomical tracing for determining neurological tracts in animal models (18, 19).

Encouraged by the preclinical data supporting axonal regeneration through PNS grafts in thoracic SCI, we set out to translate the concept of a biodegradable guiding device from the size and design required for studies in rodents to the dimensions required for a clinical study in SCI patients.

### Aim

The aim of the current study was to design a spinal cord device for guiding axons from white matter to gray matter through peripheral nerve grafts across a complete thoracic SCI in humans and to define the expected error-of-fit through simulation.

## Materials and Methods

### Data

#### Transverse and Anteroposterior Diameters of the Human Spinal Cord

We used our previously published data on the mean size and distribution estimates of the human spinal cord, as detailed in Table 1 (9). This prior study attempted to collect and summarize all known available data on the segmental size of the human spinal cord.

**Table 1:** Estimated spinal cord diameters from Frostell et al. 2016 used as a basis for simulation in the current study.

Spinal cord segment	Transverse diameter	Anteroposterior diameter	Number of subjects
C1	11.3 ± 1.7	8.3 ± 1.6	26
C2	11.5 ± 1.9	8.2 ± 1.6	181
C3	12 ± 2.3	8 ± 1.6	318
C4	12.8 ± 2.4	7.7 ± 1.7	362
C5	13.3 ± 2.2	7.4 ± 1.6	234
C6	13.1 ± 1.9	7 ± 1.6	438
C7	12.5 ± 1.9	6.9 ± 1.6	488
C8	11.3 ± 2.2	6.8 ± 1.6	336
T1	10.7 ± 2.3	6.9 ± 1.6	316
T2	10 ± 2.3	6.9 ± 1.7	27
T3	9.6 ± 2	6.8 ± 1.8	131
T4	9.5 ± 1.9	6.6 ± 1.9	131
T5	9.2 ± 2.4	6.4 ± 1.9	65
T6	8.7 ± 3	6.4 ± 1.9	65
T7	8.4 ± 2.7	6.3 ± 2	167
T8	8.3 ± 2.1	6.3 ± 2	77
T9	8.6 ± 1.7	6.5 ± 2	65
T10	8.6 ± 1.8	6.5 ± 2	65
T11	8.3 ± 2.1	6.4 ± 1.9	65
T12	8.2 ± 2.1	6.4 ± 1.8	27
L1	8.6 ± 1.9	6.7 ± 1.7	65
L2	9.1 ± 1.6	7.2 ± 1.6	27
L3	9.4 ± 1.5	7.5 ± 1.6	77
L4	9.3 ± 1.5	7.5 ± 1.6	27
L5	8.8 ± 1.7	7.1 ± 1.8	27
S1	8.4 ± 1.9	6.8 ± 2	129
S2	7.1 ± 2.5	5.8 ± 2.4	65
S3	6.3 ± 2.8	5.2 ± 2.7	27
S4	5.5 ± 3.2	4.6 ± 2.9	15
S5	4.7 ± 3.5	3.9 ± 3.2	15

*C, cervical; T, thoracic; L, lumbar; S, sacral*.

*Values represent mean ± 2 SD*.

#### White-to-Gray Matter Delineation in the Human Thoracic Spinal Cord

We used two representative series of microscopic slides covering the entire human spinal cord segmentally found in the literature (14, 15).

#### Spinal Tracts in Humans

We used schematic representations found in the literature in combination with degeneration studies to estimate the anatomical location of the spinal tracts (16, 17, 20).

### Simulation

#### Simulating a Population of Spinal Cord Sizes

To facilitate visualizations and calculations, the population estimates from Frostell et al. 2016 were used to generate a theoretical sample of spinal cords with the same mean and distribution as in the published paper. Two hundred samples per spinal cord level were generated using the mvrnorm()-function in R with the package “MASS” (21, 22). The correlation of the bivariate Gaussian distributions generated was set differently for each segment in a continuous fashion, dependent on transverse diameter. These ranged from a higher correlation (0.9) in the small segments (sacral spinal cord) to a lower correlation (0.4) in the large segments (cervical cord). This was done to satisfy the shape constraint observed in raw data of spinal cord sizes (i.e., the spinal cord can be neither too “flat” nor too “round”) (23).

Plotting was done in R using the “ggplot2” and “cowplot” packages (24). All raw data, results from simulation, and code are available upon request.

### Elliptical Interfaces

#### Determining a Set of Ellipses for Guiding Devices Covering the Thoracic Spinal Cord

Given the population estimate of the spinal cord segments T2–T12, we chose seven elliptical shapes that covered these thoracic spinal cord segments by using three different ellipses with a ratio between anteroposterior and transverse diameter (RAPT) equal to the mean RAPT of the thoracic spinal cord (termed “normal” shape), an additional two ellipses with a rounder shape than the mean RAPT (termed “round” shape), and two ellipses with a flatter shape than the mean RAPT (termed “flat” shape). The ellipses were spaced symmetrically and placed to cover the central part of the population estimates.

Another three large ellipses were added—one for each shape—to prepare for the event of spinal cord swelling due to surgical manipulation. These extra ellipses were not included in the error-of-fit calculations since they were intended as an extra safety measure.

### Error-of-Fit

#### Calculating the Error Between Thoracic Spinal Cord Segments and Guiding Devices

To calculate the error-of-fit between our set of guiding device sizes and the simulated spinal cord sizes, we assigned each simulated spinal cord segment (between T2 and T12) to the best-fitting guiding device in our set of seven sizes. The best-fitting guiding device was chosen by attempting to minimize both transverse and anteroposterior difference between the simulated segment and the elliptical shape of the guiding device. This was accomplished by minimizing the square root of the sum of the squares of the transverse and anteroposterior error (equal to minimizing the Euclidian distance between the simulated spinal cord segment and the guiding device in a two-dimensional space representing transverse and anteroposterior diameter). After the best-fitting guiding device was chosen for every simulated spinal cord size, we calculated the transverse and anteroposterior error separately, as well as the mismatch in area. This was done for every simulated spinal cord segment, which resulted in 200 segments per level between T2 and T12 for a total of 2,200 simulated spinal cord sizes.

### Spinal Cord Guiding Device

#### Creating a Vector Model of the Human Spinal Cord

Images covering the entire human spinal cord were available in a digital format and were imported in a CAD program (Rhinoceros for Mac, version 5). The outline of the spinal cord and the delineation between white and gray matter were traced manually to create a vector model. The midline of the spinal cord was identified, and both traced halves were averaged to yield symmetric white-to-gray matter delineations for each segmental level of each spinal cord series. Furthermore, each segmental image was scaled to the average transverse and anteroposterior diameter for that segment using the data collected and presented in an earlier publication (Frostell et al. 2016). Thereafter, segments T2–T12 were averaged in both spinal cord series, and both thoracic averages were combined into a final average representation of the delineation between white and gray matter in the human thoracic spinal cord.

#### Designing the Guiding Device Interfaces and Channels

The device was designed in a CAD program (Rhinoceros for Mac, version 5). The guiding channels of the device were chosen using the vector model of the spinal cord and anatomical knowledge of the spinal cord tracts in humans. Channels were designed to start at a relevant descending (e.g., corticospinal tract) or ascending tract (e.g., dorsal column) and run obliquely through the device to reach gray matter at the other side of the device. Channels were spaced so as not to intersect each other and to leave a wall of material of at least 0.4 mm between channels.

#### Assessing the Alignment of Guiding Device Channels and Spinal Cord White Matter Tracts

To assess the alignment of the device channels, the vector model of the average thoracic spinal cord and the guiding device interfaces were imported in R. The spinal cord shape was then scaled to the dimensions of each simulated thoracic spinal cord segment and overlaid on the shape of the device that showed the best fit for that particular simulated spinal cord segment. The results were assessed qualitatively.

#### Determining the Final Set of Guiding Devices

From the design process described above, we choose 7 guiding device sizes and an extra 3 larger sizes for a total of 10 interface sizes. Because the spinal cord is mobile to some extent in the spinal canal, the length of the guiding device was chosen with 5-mm increments from 15 to 40 mm, covering the shorter range of thoracic SCIs (8). This resulted in a total of 60 guiding devices (10 interfaces × 6 lengths).

## Results

### Simulation

#### Simulated Segmental Spinal Cord Sizes

**Figure 1:**
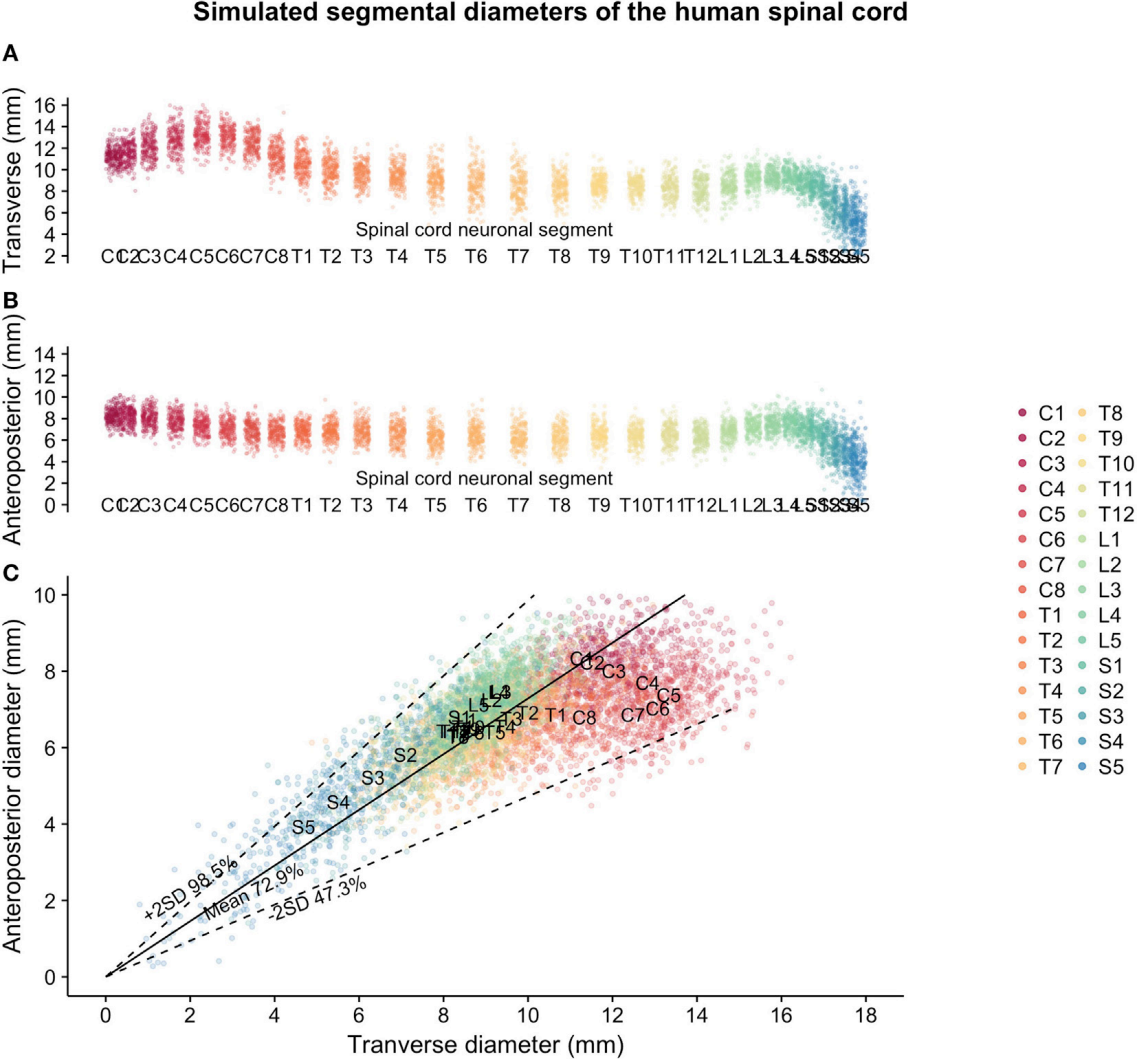
Simulated spinal cord sizes based on population estimates from Frostell et al. 2016: **(A)** transverse diameter of the human spinal cord per neuronal segment, **(B)** anteroposterior diameter of the human spinal cord per neuronal segment, and **(C)** relationship between transverse and anteroposterior diameters of the human spinal cord.

**Figure 2:**
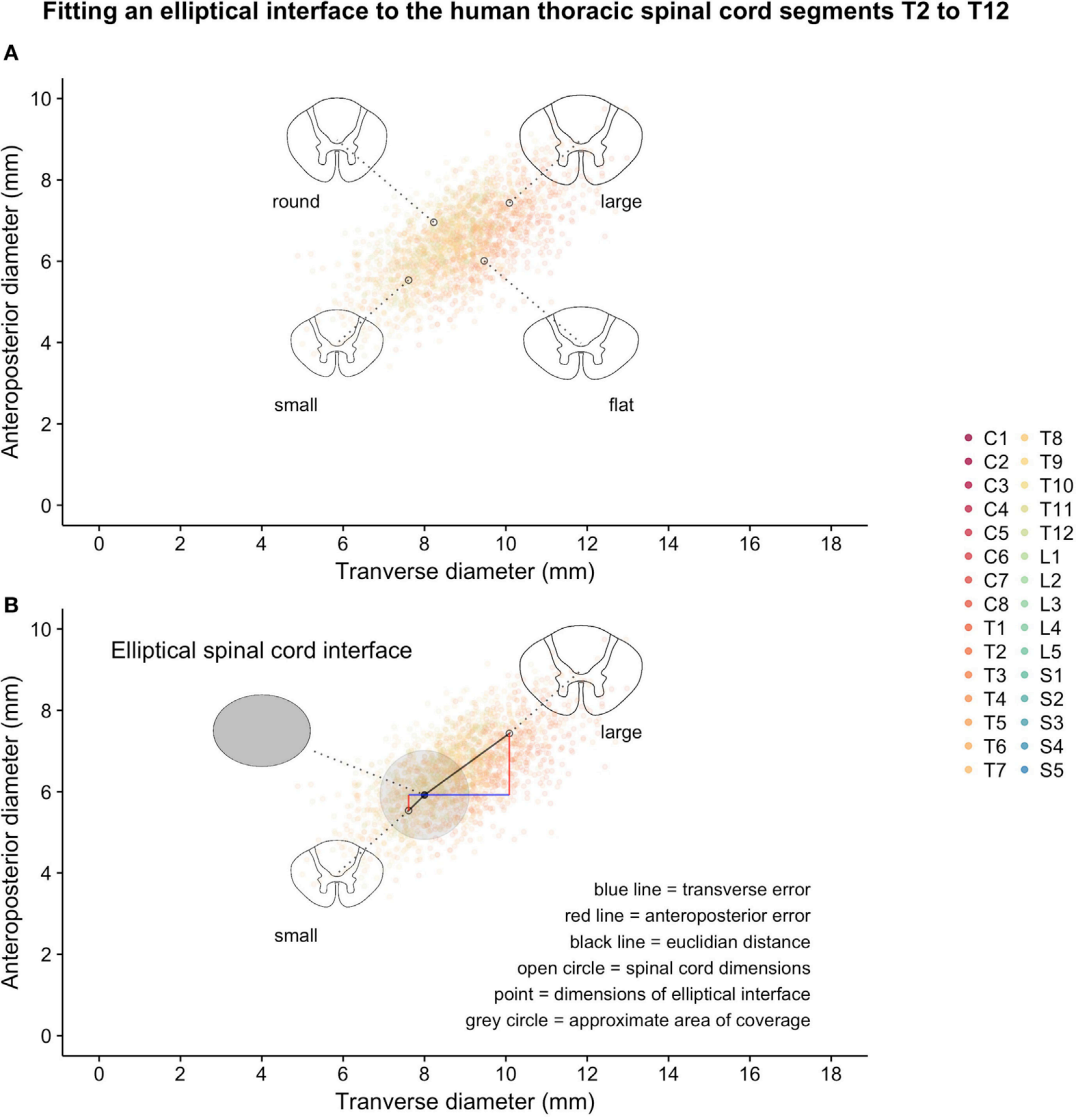
Thoracic segments T2–T12 from the same simulated data shown in Figure 1: **(A)** examples of possible shapes and sizes of the thoracic spinal cord and **(B)** introduction of a hypothetical elliptical spinal cord interface and geometrical representations of various measures of error-of-fit as calculated.

Two hundred matched spinal cord transverse and anteroposterior diameters were simulated for each segmental level of the human spinal cord based on data from Frostell et al. 2016. Varying the correlation of the bivariate distribution for each segmental level (from a higher correlation in the lumbar spine to a lower correlation in the cervical spine) allowed the simulated data to satisfy not only the targeted distribution of transverse and anteroposterior diameters but also the shape constraint observed in raw data from measurements of real patients (i.e., regardless of variations in size, the cross section of the spinal cord can never be too “round” nor too “flat”). Figure 1 shows the simulation of spinal cord sizes along the spinal cord. From the results obtained, the variation between segments of the spinal cord’s elliptical shape and size was much smaller in the thoracic cord compared to the cervical and lumbar portions of the spinal cord. The variation likely to occur in the thoracic spinal cord segments T2–T12 is shown in Figure 2A, along with correctly scaled images visualizing the size and shape of the segmental anatomy.

### Error-of-Fit

#### Error Between the Simulated Thoracic Spinal Cord and Guiding Devices

**Figure 3:**
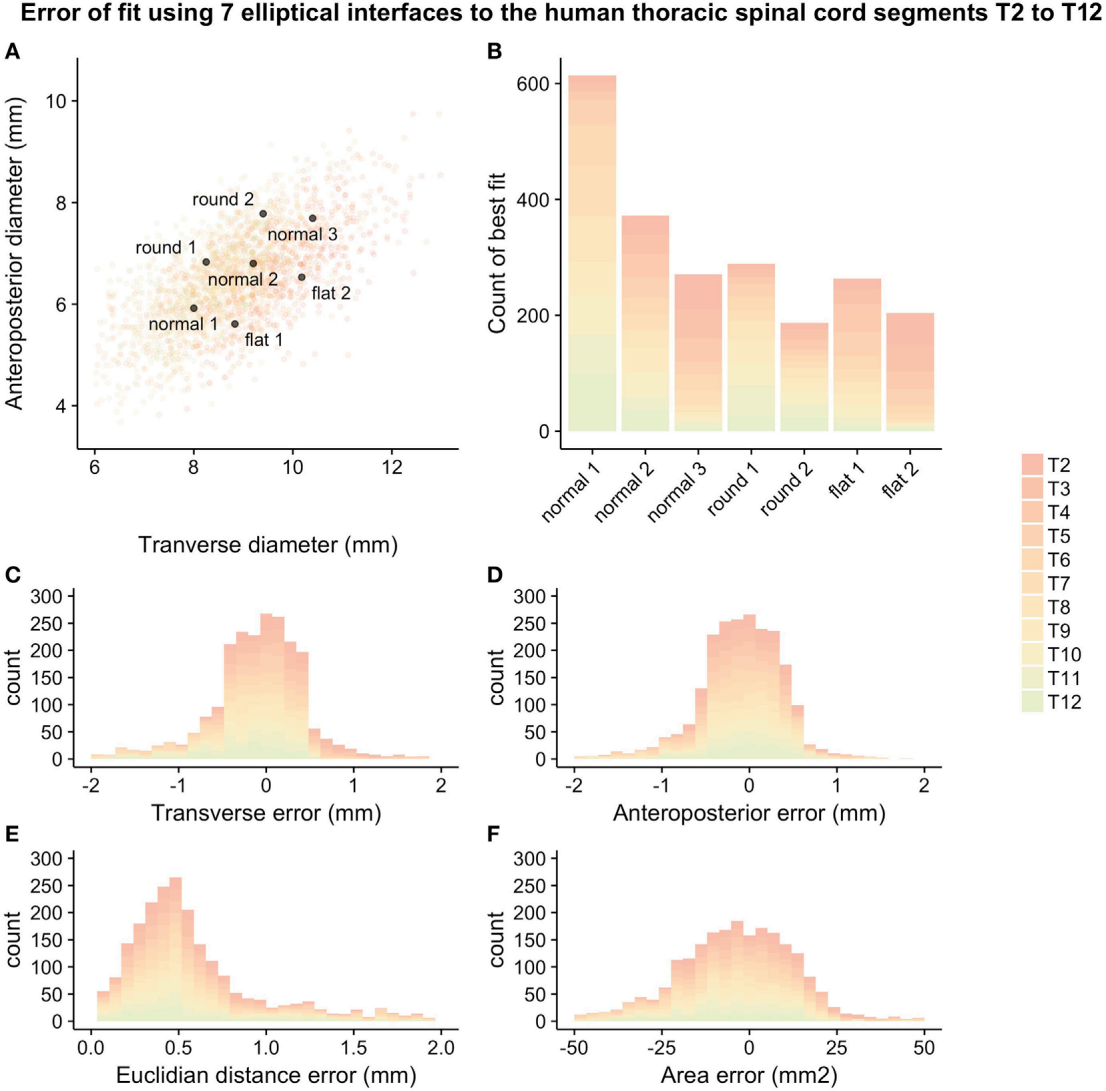
Error-of-fit between simulated thoracic spinal cord segments T2–T12 and guiding device sizes represented by seven ellipses: **(A)** overlay of device sizes and simulated data, **(B)** best-fitting device for the simulated data, **(C)** transverse error-of fit, **(D)** anteroposterior error-of-fit, **(E)** Euclidian distance error, and **(F)** area error. For numerical values of the different measures of error, see Table 2.

The chosen set of seven guiding device sizes was compared to the simulated thoracic segments T2–T12. For each simulated thoracic segment, we calculated the Euclidian distance to the guiding device sizes as detailed in Figure 2B to find the device that best fit a given simulated thoracic spinal cord segment (Figures 3A,B). The guiding device size termed “normal 1” was the most frequent “best fit,” reflecting its centered position over the distribution of thoracic spinal cord sizes and the design choice to focus on having enough head room to handle intraoperative swelling of the spinal cord due to manipulation (Figure 3B).

The mean error-of-fit comparing simulated spinal cord segments T2–T12 to the best elliptical shape was 0.41 and 0.36 mm, the median was 0.31 and 0.31 mm, and the 95th percentile was found at 1.3 and 0.98 mm for transverse and anteroposterior diameter, respectively. The mean, median, and 95th percentile of the Euclidian distance was 0.60, 0.48, and 1.63, respectively. The mean and median area mismatches were 14.23 and 10.93 mm^2^, respectively, and the 95th percentile was found at 41.40 mm^2^.

Figure 3 shows all seven ellipses as well as a graphical representation of the different measures of error-of-fit that were calculated. Table 2 shows the mean, median, and 95th percentile of the different measurements of error-of-fit.

**Table 2:** Calculated measures of error-of-fit between simulated spinal cord segmental diameters in segments T2–T12 and the chosen set of seven guiding device sizes.

	Median error	Mean error	95th percentile
Euclidian distance	0.48 mm	0.60 mm	1.63 mm
Transverse error	0.31 mm	0.41 mm	1.30 mm
Anteroposterior error	0.31 mm	0.36 mm	0.98 mm
Area mismatch	10.93 mm^2^	14.23 mm^2^	41.40 mm^2^

### Design of the Guiding Device

#### The Vector Model of the Human Thoracic Spinal Cord

**Figure 4:**
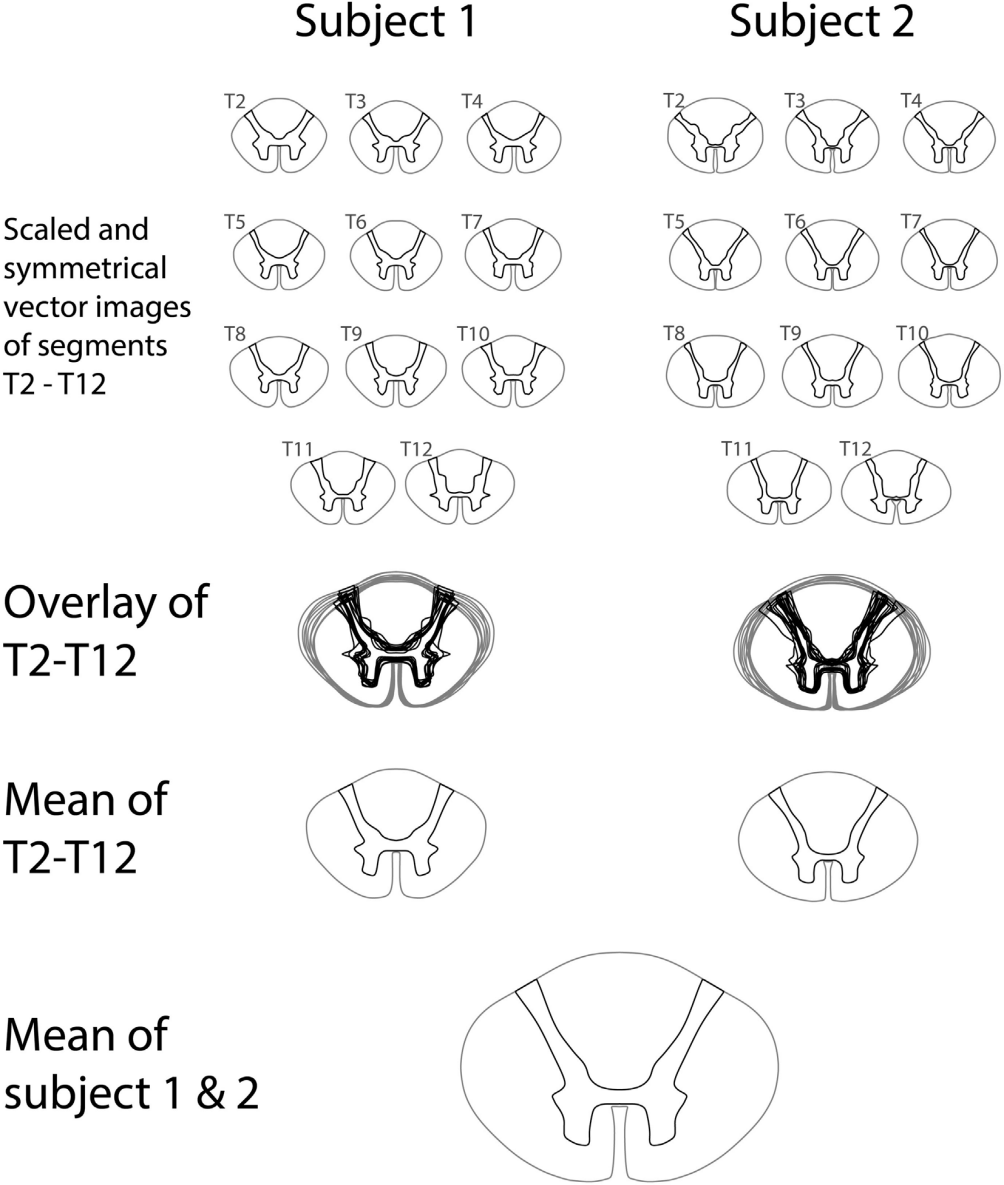
Construction of a vector representation of the average thoracic spinal cord shape and white-to-gray matter delineation by averaging segments T2–T12 from two published series of spinal cord segments.

The variations in white-to-gray matter delineation in segments T2–T12 were small both within subjects and between subjects. The angle of the dorsal horn increased in the cranial direction in both subjects, and the transverse distance between the anterior gray matter on each side was slightly different between subjects. Figure 4 shows the steps involved in constructing a vector model of the mean thoracic spinal cord shape in segments T2–T12 by combining shapes from all thoracic spinal cord segments from two published sources of the human spinal cord.

#### The Spinal Cord Interfaces and Graft Channels of the Guiding Device

**Figure 5:**
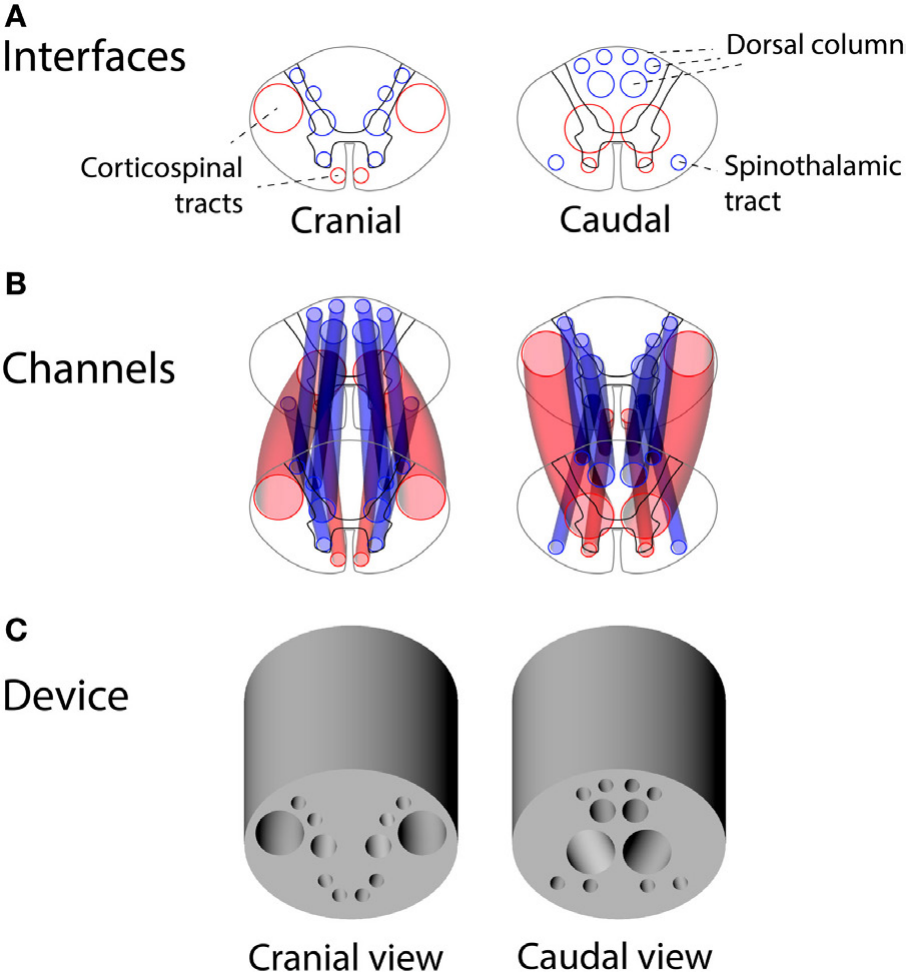
Design of the guiding device for precision grafting of peripheral nerves in thoracic spinal cord injury: **(A)** cranial and caudal interfaces based on human spinal tracts and white-to-gray matter rerouting of axons, **(B)** a 3D overview of guiding channels satisfying constraints of non-intersecting channels and minimal wall thickness of 0.4 mm, and **(C)** a 3D rendering of the guiding device.

Using the vector model of the human thoracic spinal cord, we designed a set of channels connecting the two spinal cord interfaces with each other for placement of the autologous peripheral nerve grafts. A device design was reached for capturing the majority of corticospinal axons at the cranial interface of the device and guiding them obliquely to gray matter at the caudal interface. Figure 5 shows the interfaces of the guiding device alongside the placement of the channels for peripheral nerve grafts guiding axons from white-to-gray matter across a complete SCI.

#### The Alignment of Guiding Device Channels and Spinal Cord White Matter Tracts

**Figure 6:**
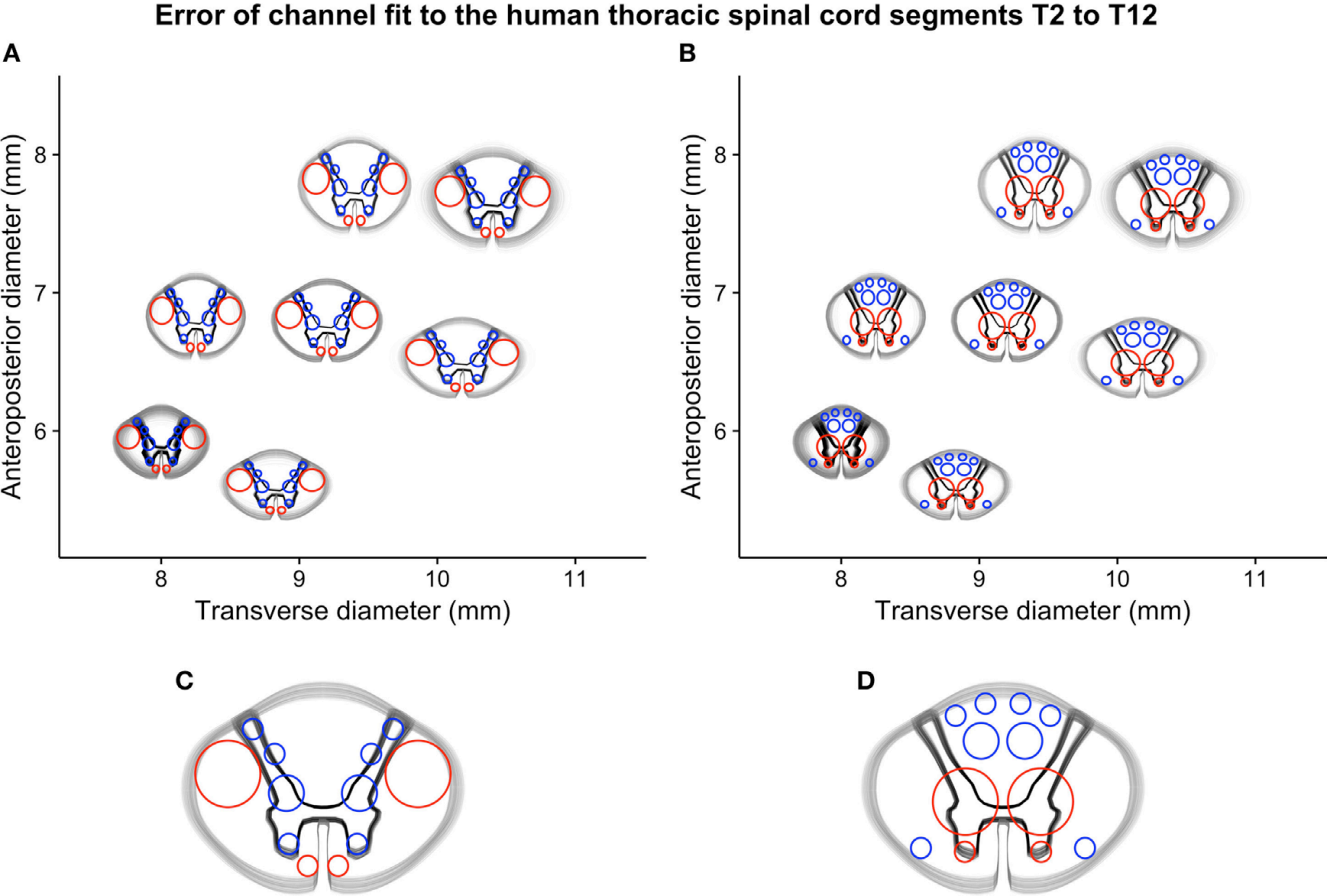
Simulated error-of-fit of guiding channels to thoracic spinal cord segments T2–T12: **(A)** all segments fitted to the best-fitting cranial interface, **(B)** all segments fitted to the best-fitting caudal interface, and **(C)** and **(D)** a detailed representation of both interfaces of the mid-sized guiding device (termed “normal 2”), showing the small deviation between guiding channels and intended position on the spinal cord interfaces.

By comparing the white-to-gray matter delineation from the vector model scaled to the sizes of the simulated spinal cord segments and overlaid with the best-fitting device size, we made a qualitative assessment of the alignment between the guiding device channels and the spinal cord white matter tracts. This alignment was considered satisfactory except in the most extreme cases and is shown in Figure 6.

## Discussion

Encouraged by the preclinical data supporting axonal regeneration through PNS grafts in SCI (2–6), we set out to translate the concept of a biodegradable guiding device from the size and design required for studies in rodents to the dimensions required for a clinical study in SCI patients.

To allow measurement and visualization of the error-of-fit for guiding device size and channel interfaces, a population of 200 spinal cords was generated through simulation. This approach relies on the extrapolation of spinal cord size from a healthy population to the SCI population. In the chronic phase of SCI, the SCA at the C2 vertebral level (cranial to the injury) has been shown to decrease (10–13). The majority of patients investigated in these studies had cervical injuries. In the only study that included a significant number of thoracic injuries (13), cervical injuries decreased more than thoracic injuries in SCA compared to controls. The mechanism for this decrease in SCA has not been fully uncovered, and unfortunately none of these prior studies measured the SCA at the thoracic level. Therefore, it is not entirely clear how to apply this knowledge when estimating the spinal cord dimensions at the thoracic level in a patient suffering from a complete thoracic SCI. We did not compensate for this possible decrease in size of the thoracic spinal cord in SCI patients for the design and sizing of the guiding device; however, since our approach defines a set of devices with different shapes covering the range of possible sizes in the normal population, we believe that adequate fit would be obtained even in the event of moderate atrophy of the thoracic spinal cord, as has been described in the cervical spinal cord in chronic SCI.

The primary numerical error-of-fit investigated was the Euclidian distance from the simulated thoracic spinal cord sizes (T2–T12) to the best-fitting device, thereby combining the error-of-fit of the transverse and anteroposterior diameter. With the simulated data and the seven guiding device dimensions chosen, the median error-of-fit was about 0.5 mm, and the most extreme sizes (95% percentile) showed an error-of-fit of about 1.5 mm. This number signifies the total difference in width between the device and the spinal cord. It gives an overall estimate of the error-of-fit but should be considered a proxy measure for the channel-to-spinal-tract fit and the microsurgical precision in placement and fixation of the device to the spinal cord during surgery. The actual error between a guiding channel and the intended anatomical position on the spinal cord will always be smaller than the Euclidian distance for the following reasons: (1) the error is divided symmetrically between the halves of the spinal cord and (2) the error will be further reduced relative to the distance from the midpoint of the device (i.e., objects close to the midpoint will be correctly positioned regardless of the difference in size between the spinal cord and the guiding device). We also added three extra-large guiding device sizes that were not included in the calculations as a safety measure in the event of swelling of the spinal cord.

Qualitative assessment of the channel-to-spinal-tract fit using an overlay of each of the 200 simulated spinal cord sizes for every thoracic segment (T2–T12) with the best-fitting device showed a satisfactory alignment of spinal cord anatomy and device channels for the simulated spinal cords. However, the precision required for successful spinal cord regeneration is unknown, and the choice of 7 + 3 device sizes represents our best estimate.

Channel placement and design were based on the concept of white-to-gray matter guiding through peripheral nerve grafts as defined by Cheng et al. (3), refined by Nordblom et al. (5, 6), and adapted to the dimensions of the human spinal cord and the location of the spinal tracts found in humans (9, 17, 20). The design prioritized allowing the lateral corticospinal tract at the cranial interface to connect to gray matter at the caudal interface. Unfortunately, for obvious technical reasons, data on human spinal tracts lack the detail available in studies using experimental animals. Hence, it is difficult to calculate an exact measurement of the error-of-fit of the guiding device channels in relation to the spinal cord anatomy.

This study details the design and sizing of an SCI repair device for exact microsurgical placement of PNS grafts to promote the regeneration of axons from white-to-gray matter across a thoracic (T2–T12) complete (AIS-A) SCI currently in use in a clinical trial (http://ClinicalTrials.gov identifier: NCT02490501).

## Conclusion

Detailed knowledge of the three-dimensional anatomy of the human spinal cord and its variation is required for the design of a guiding device for spinal cord regeneration. A set of guiding device interfaces of seven sizes can cover the variability of human thoracic spinal cord segments T2–T12 with an acceptable error-of-fit for the elliptical shape as well as guiding channels. In studies where a premade device, instrumentation, or other physical object needs to be applied to the spinal cord, the conceptual framework described in this paper will be relevant.

## Ethics Statement

This paper was based solely on published papers and their reported data. We did not conduct any radiological or postmortem examinations. This paper itself is exempt from ethical approval but relies in part on the ethics of the included studies, which we have found no reason to question.

## Author Contributions

Substantial contributions to the conception or design of the work (AF, PM, and MS) and the acquisition (AF), analysis (AF), or interpretation (AF, PM, and MS) of data for the work. Drafting the work or revising critically for important intellectual content (AF, PM, and MS). Final approval of the version to be published (AF, PM, and MS). Agreement to be accountable for all aspects of the work in ensuring that questions related to the accuracy or integrity of any part are appropriately investigated and resolved (AF, PM, and MS).

## Conflict of Interest Statement

The authors wish to declare a conflict of interest due to their role as inventors on patent applications regarding the spinal cord injury device described in the paper (US 20090169596 A1, authors MS and PM; US20150088257 A1, all authors). The company BioArctic AB controls all patent rights through a direct ownership or an exclusive licensing, and the company has provided funding for the study and approved the manuscript in its present form. The authors do not control or have ownership in BioArctic AB. Senior authors MS and PM also appear on the scientific advisory board of BioArctic AB. Furthermore, the senior author MS is the principal investigator of a clinical trial using the described device (http://ClinicalTrials.gov identifier: NCT02490501). The clinical trial is funded in part by BioArctic AB and has also received funding from the European Union’s Horizon2020 Research and Innovation Program under Grant Agreement No. 643853 to perform a clinical study.

## Abbreviations

SCI, spinal cord injury; CNS, central nervous system; PNS, peripheral nervous system; MRI, magnetic resonance imaging.
